# Pharmacologically targeting Schwann cells to improve regeneration following nerve damage

**DOI:** 10.3389/fcell.2025.1603752

**Published:** 2025-06-19

**Authors:** Alaa A. Alhamdi, Shona Mackie, Ryan P. Trueman, Melissa L. D. Rayner

**Affiliations:** ^1^ Department of Pharmacology, UCL School of Pharmacy, University College London, London, United Kingdom; ^2^ UCL Centre for Nerve Engineering, University College London, London, United Kingdom

**Keywords:** nerve regeneration, Schwann cells, signalling pathway, pharmacological intervention, cell phenotype

## Abstract

Schwann cells provide essential support for nerve growth and survival following peripheral nerve damage by producing various growth factors and other signalling molecules. Modulating the proliferation, differentiation, migration, or myelination of Schwann cells could result in accelerated repair and regeneration of injured nerves, ultimately leading to improved motor and sensory function. Therefore, Schwann cells are commonly advocated as therapeutic targets for nerve damage, which could be mediated by pharmacological intervention. This review discusses how compounds such as growth factors, hormones, and small molecules can regulate intracellular signalling pathways involved in modulating Schwann cells.

## 1 Introduction

The intricate process of nerve regeneration relies on the crucial role played by Schwann cells, the principal glial cells of the peripheral nervous system (PNS) (Jessen et al., 2015). Schwann cells exhibit remarkable plasticity, displaying distinct phenotypic characteristics in response to different cues within their microenvironment (Jessen and Mirsky, 2016; Kim et al., 2017). Manipulating Schwann cell phenotype has emerged as a promising strategy to enhance the rate of nerve regeneration, offering potential therapeutic applications in treating nerve injuries and neuropathies (McMorrow et al., 2022). This review aims to comprehensively explore pharmacological therapies that have shown to alter Schwann cell phenotype. By shedding light on these advancements, we hope to contribute to the development of innovative therapeutic approaches to promote nerve regeneration.

Healthy peripheral nerves contain two distinct Schwann cell phenotypes: myelinating and non-myelinating Schwann cells (Remak cells) (Jessen and Mirsky, 2019). These two Schwann cell populations exhibit distinct morphological and functional characteristics. Remak Schwann cells encircle multiple axons with diameters of <1 μm, while myelinating Schwann cells ensheath individual axons with larger diameters, wrapping around the axon multiple times to form a densely packed myelin sheath (Harty and Monk, 2017; Michailov et al., 2004). Both Remak and myelinating Schwann cells play an important role in providing metabolic and trophic support to the neuron (Jessen and Mirsky, 2005; Vega et al., 2003); however, the primary functional distinction between the two Schwann cell types is that myelinating Schwann cells enhance nerve impulse firing and conduction through saltatory conduction facilitated by the myelin sheath (Jessen and Mirsky, 2019). These two categories of Schwann cells can be further distinguished by their distinct protein expression profiles (Jessen and Mirsky, 2016; Jessen and Mirsky, 2005). Remak cells share markers expressed by immature Schwann cells, such as neural cell adhesion molecule (NCAM), p75 neurotrophin receptor (p75^NTR^), glial fibrillary acidic protein (GFAP), and L1 cell adhesion molecule (L1CAM) (Jessen and Mirsky, 2019). Conversely, myelin Schwann cells express an array of proteins associated with the formation and maintenance of the myelin sheath, including protein zero (P0), myelin basic protein (MBP), and myelin-associated glycoprotein (MAG) (Jessen et al., 2015).

Schwann cells play a critical role in the regeneration of injured peripheral nerves by promoting axonal regrowth and remyelination. Mature Schwann cells possess the remarkable ability to undergo phenotypic reprogramming following damage, becoming more pro-regenerative (Jessen and Mirsky, 2016; Jessen and Mirsky, 2019; Nocera and Jacob, 2020). Following injury, the nerve distal stump undergoes Wallerian degeneration (Arthur-Farraj et al., 2012), and Schwann cells transition from their myelinating phenotype to a repair-promoting phenotype by downregulating myelin-associated proteins such as P0 and MBP, and upregulating genes that support regeneration, including c-Jun, activating transcription factor 3 (ATF3), growth-associated protein 43 (GAP-43), and sonic hedgehog (Shh) (McMorrow et al., 2022). Persistent expression of myelin-associated proteins has been shown to inhibit the full activation of the repair program in Schwann cells, thereby limiting their capacity to support axonal regeneration (Nguyen et al., 2009; Filbin, 1995).

The repair Schwann cells proliferate, while secreting cytokines such as tumor necrosis factor-α (TNF-α), leukemia inhibitory factor (LIF), and interleukin-1β (IL-1β) to recruit immune cells including macrophages to aid in myelinophagy and cellular debris clearance (Jessen and Mirsky, 2016; Rotshenker, 2011). The M2 macrophages also secrete a lysosomal cysteine protease, cathepsin S (CTSS) which degrades extracellular matrix (ECM) proteins, restructuring the nerve microenvironment to allow for cellular migration and promote regeneration (Oshima et al., 2023). The repair Schwann cells elongate to 2-3 times the length of mature Schwann cells (Gomez-Sanchez et al., 2017), and secrete neurotrophic factors and extracellular growth cues, including glial cell line-derived neurotrophic factor (GDNF), brain-derived neurotrophic factor (BDNF), neurotrophin-3 (NT3) and nerve growth factor (NGF), which promote axonal survival and regeneration (Jessen and Mirsky, 2019). Following Wallerian degeneration, the repair Schwann cells align themselves within cellular columns known as the bands of Büngner, providing guidance cues as well as physical support to regenerating axons (Gomez-Sanchez et al., 2017). M2 macrophage-derived CTSS activate nearby fibroblasts to support regeneration by secreting pro-regenerative factors and ECM glycoproteins such as tenascin-C, which bind to β1 integrins on Schwann cells and promote their directed migration during the formation of the bands of Büngner (Oshima et al., 2023; Zhang et al., 2016). The transition from a myelinating Schwann cell to the repair phenotype is reversible and critical to effective nerve repair. Once axonal regrowth has progressed and contact is re-established, repair Schwann cells revert to their myelinating phenotype, re-expressing myelin-associated genes to support functional recovery (Jessen and Mirsky, 2008; Fex Svennigsen and Dahlin, 2013).

In addition to interacting with neurons, macrophages, and fibroblasts, Schwann cells engage in dynamic crosstalk with other cell types during nerve regeneration. Recent findings demonstrate that adipocytes support Schwann cell-mediated nerve regeneration by promoting mitochondrial metabolism through leptin receptor signalling. This adipo-glial interaction facilitates the metabolic reprogramming essential for Schwann cell repair function following PNI (Sundaram et al., 2023).

Despite Schwann cells providing a supportive environment, challenges in obtaining successful nerve regeneration persist. Axonal regeneration is rate-limited with axon regrowth being limited to ∼1 mm per day in humans (Grinsell and Keating, 2014). This slow rate of regeneration frequently results in chronic denervation of the distal nerve segment and end-target organ, due to the lack of timely reinnervation (Scheib and Hoke, 2013). Chronically denervated Schwann cells contribute to this outcome in two manners. Firstly, without axonal contact, the transient growth-permissive repair phenotype of Schwann cells is lost over time, resulting in a decrease in neurotrophin expression and thus a reduced regenerative capacity. Secondly, Schwann cell apoptosis in the distal stump follows the loss of their repair phenotype, resulting in the deterioration of the bands of Büngner (Vuorinen et al., 1995; Sulaiman and Gordon, 2000). After 2–3 months without axonal contact, the number of Schwann cells decreases by 30%–50% (Jessen and Mirsky, 2019), contributing to the distal segment’s reduced capacity to support regeneration (Sulaiman and Gordon, 2000; Wagstaff et al., 2021). Therefore, the gradual loss of the repair phenotype, coupled with the eventual decrease in Schwann cell number, are likely the dual factors contributing to the diminished regeneration in chronically denervated stumps.

The reprogramming of Schwann cells into their repair state is primarily orchestrated by nuclear transcription factors. In particular, the transcription factor c-Jun is a key driver of the Schwann cell repair phenotype (Jessen and Mirsky, 2016), which is rapidly upregulated following injury (Stewart, 1995). Activation of c-Jun occurs downstream of various mitogen-activated protein kinase (MAPK) signalling pathways, such as JNK, ERK1/2, and p38 (Yang et al., 2012; Boerboom et al., 2017; Balakrishnan et al., 2020) and the activation of mTOR complex 1 (mTORC1), downstream of the PI3K pathway, has been identified as a critical factor in initiating the repair phenotype (Norrmen et al., 2018). c-Jun exerts its influence on more than 170 genes and primarily contributes to the promotion of the Schwann cell repair phenotype by functioning as a negative regulator of myelination. It achieves this by suppressing the expression of key myelination-related components such as the transcription factor Krox20 (also known as early growth response 2 (Egr2)), as well as proteins including P0 and MBP (Arthur-Farraj et al., 2012; Parkinson et al., 2008). Furthermore, c-Jun regulates genes which facilitate myelin degradation, and activates a wide array of repair-promoting characteristics, such as the increased expression of neurotrophins (Jessen and Mirsky, 2016; Arthur-Farraj et al., 2012). Notably, c-Jun increases the expression of several signalling molecules and trophic factors including Shh protein, GDNF, neuregulin 1 (NRG1) type 1, insulin-like growth factor 1 (IGF-1) and transforming growth factor β (TGFβ). These signals act through autocrine loops to further promote the repair-associated activity in Schwann cells (Jessen and Mirsky, 2021). As time progresses, the expression of c-Jun gradually diminishes in chronically denervated Schwann cells. By 10 weeks post-injury, c-Jun declines to approximately 40%–50% of the expression levels observed during the initial 1–2 weeks post-injury (Wagstaff et al., 2021; Wilcox et al., 2021). Maintaining or increasing c-Jun expression has therefore emerged as a promising strategy for preserving the repair phenotype of chronically denervated Schwann cells.

Transcription factors SOX2 and SOX10 are also known to play essential roles in Schwann cell differentiation and myelination. Like c-Jun, SOX2 has been identified as a negative regulator of myelination (Le et al., 2005). SOX2 is also linked to pluripotency and stemness in different cell lineages and is downregulated by the myelination-associated transcription factor Krox20 (Egr2) (Le et al., 2005). Additionally, SOX2 is involved in Schwann cell migration and formation of the bands of Büngner, mediating directional organization through ephrin-B/ephB2 signalling (Parrinello et al., 2010). On the other hand, SOX10 is a crucial transcription factor for Schwann cell development from the neural crest (Jessen and Mirsky, 2005; Bhatheja and Field, 2006). Contrary to SOX2, SOX10 is involved in activating myelin gene transcription in coordination with Krox20 (Egr2), binding to enhancer sites in myelin-associated genes (Hung et al., 2015). Interestingly, although SOX10 is critical for Schwann cell development, its expression remains unchanged after a peripheral nerve injury (PNI) (Arthur-Farraj et al., 2012). Due to its function as a positive regulator of myelination, SOX10 inhibition offers a potential avenue for intervention to promote the repair-associated characteristics of Schwann cells (Aberle et al., 2024). Conversely, expression of SOX2, a factor that inhibits myelination, could be elevated to maintain regeneration-supportive features of Schwann cells.

Signal transducer and activator of transcription 3 (STAT3) is another transcription factor that has been implicated in the Schwann cells’ response to injury. STAT3 activation is crucial for the long-term maintenance of the repair phenotype (Benito et al., 2017). Furthermore, STAT3 has been shown to enhance Schwann cell survival as well as modulate proliferation and migration (Benito et al., 2017; Chen et al., 2021). Together, these findings highlight the multifunctional role of STAT3 in the Schwann cells’ response to PNI, encompassing their survival, maintenance, and dynamic behaviours. However, like c-Jun, STAT3 activation decreases during chronic denervation, leading to a decline in the repair Schwann cell phenotype. This reduction in STAT3 activity impairs Schwann cell support for regeneration, as repair-associated functions and the expression of trophic factors are diminished (Jessen and Mirsky, 2016; Benito et al., 2017).

In the later stages of regeneration, once axonal contact with the target tissue has been re-established, remyelination is critical for restoring nerve function. Liver kinase B1 (LKB1) is a key regulator of Schwann cell polarity, differentiation, and initiation of remyelination following PNI (Beirowski et al., 2014). Activation of adenosine monophosphate-activated protein kinase (AMPK) downstream of LKB1 regulates several processes including lipid metabolism and mitochondrial biogenesis which are essential for myelin formation (Beirowski et al., 2014). AMPK activation also inhibits mTORC1 activity, preventing excessive proliferation and maintaining the structural and functional integrity of Schwann cells (Norrmen et al., 2018).

Recent advancements in our understanding of the complex molecular pathways regulating Schwann cell plasticity have highlighted the potential of targeting phenotypic modulation as a strategy for treating PNI. By leveraging these mechanisms, the potential to promote regeneration and improve outcomes after injury could be realised.

## 2 Pharmacological therapies targeting Schwann cells

**FIGURE 1:**
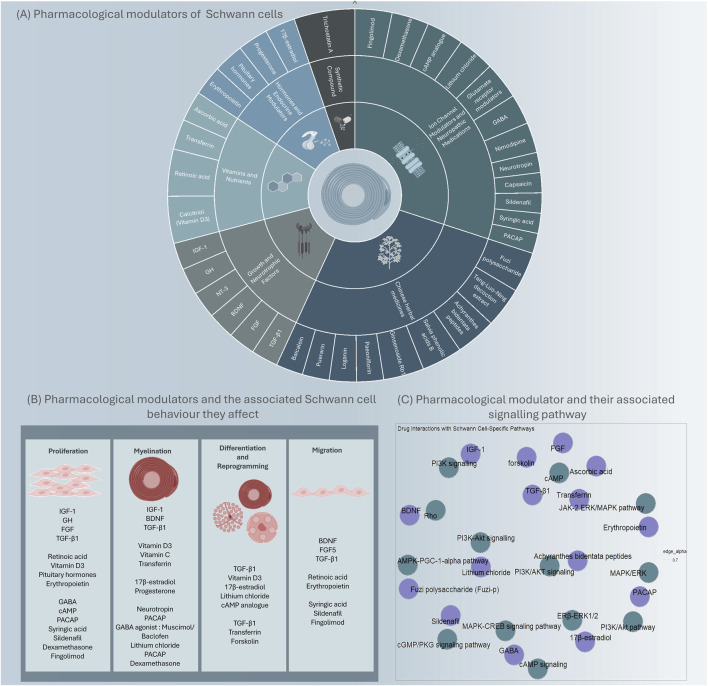
An overview of identified pharmacological modulators and key targets that could modulate Schwann cells. Modulators that have demonstrated promise in modulating Schwann cell phenotype or behaviour, are grouped by their class **(A)** and the Schwann cell behaviour they affect **(B)**. The modulators (purple) and the signalling pathway they interact with (green) **(C)**.

Pharmacological therapies offer a conventional approach to modulate Schwann cell phenotype. Compounds such as growth factors, hormones, and small molecule agonists or antagonists can be utilised to regulate intracellular signalling pathways involved in Schwann cell differentiation and myelination. For example, the administration of neurotrophic factors, such as NGF, BDNF, or GDNF, can promote the transition of Schwann cells toward a myelinating phenotype and enhance axonal regrowth (Magnaghi et al., 2009). Additionally, small molecules targeting specific receptors or downstream effectors, for example, forskolin or rolipram, have shown promising effects in inducing the expression of pro-regenerative factors and promoting nerve regeneration (Udina et al., 2010; Yamada et al., 1995). Compounds that have been studied for Schwann cell modulation will be discussed further and are summarised in Figure 1.

### 2.1 Growth and neurotrophic factors

#### 2.1.1 Insulin like growth factor-1 (IGF-1)

An accumulating number of studies have highlighted the beneficial effect of insulin-like growth factor 1 (IGF-1) treatment on Schwann cells by maintaining their survival, proliferation, maturation, differentiation and myelination (Tuffaha et al., 2016). Immunofluorescence staining confirmed the expression of the IGF-1 receptor on the surface of Schwann cells in primary rodent cells. Combining low concentrations of IGF-1 with forskolin or di-butyryl cyclic AMP (dbCAMP), resulted in improved Schwann cell growth, indicating a synergistic effect (Schumacher et al., 1993). Furthermore, within an *in vitro* co-culture of dorsal root ganglion (DRG) neurons and Schwann cells and *in vivo* using IGF-1 transgenic mice, it was demonstrated that IGF-1 upregulates the *de novo* fatty acid production by Schwann cells throughout the myelination process in a PI3K-dependent manner (Russell et al., 2000; Liang et al., 2007; Ye et al., 1995).

Furthermore, in a rat sciatic nerve crush injury model, both IGF-1 and IGF-2 mRNA expression levels were upregulated distally, decreasing as the axon regenerated, however, the same was not seen in a transection nerve injury without regeneration (Glazner et al., 1994). In another study, when Schwann cells were treated with IGF-1, IGF-2, and insulin, the production of P0 was increased, suggesting their role in promoting Schwann cell myelination (Sullivan et al., 2008; Stewart et al., 1996).

Upregulation of IGF-1 has also been shown to protect Schwann cells from glucose-induced apoptosis, both in purified Schwann cells *in vitro* and within a streptozotocin-treated diabetic rat model, suggesting a therapeutic potential of IGF-1 in diabetic neuropathy (Delaney et al., 2001). Additionally, Schwann cell mitogenesis has shown to be enhanced by IGF-1 *in vitro*, through activating the synthesis and proliferation of DNA (Stewart et al., 1996).

#### 2.1.2 Growth hormone (GH)

Growth hormone (GH) is produced by the pituitary gland and acts via IGF-1 to promote cell growth, prevent apoptosis, and enhance regeneration. Previous work suggested that GH therapy improves functional recovery following nerve lesions; an effect suggested to be due to enhancing Schwann cell proliferation (Devesa et al., 2012). Histological analysis following a sciatic nerve transection and primary repair model revealed that GH treatment elevated the expression of the Schwann cell marker S100. Additionally, the axon diameter and thickness of the myelin sheath were improved post-GH treatment in comparison to the saline control (Devesa et al., 2012).

In a study with Sprague-Dawley rats undergoing a sciatic nerve transection and repair and femoral nerve transection without repair, daily subcutaneous administration of GH was found to improve Schwann cell proliferation in the distal femoral nerve (Tuffaha et al., 2016). Evidence suggests that it could be beneficial to further explore the impact of GH and IGF-1 following PNI. Since both are already approved by the U.S. Food and Drug Administration (FDA), their clinical translation could be escalated.

#### 2.1.3 Neurotrophin-3 (NT-3)

Neurotrophins are a family of growth factors that encompass NGF, BDNF, NT-3, and neurotrophin-4/5 (NT-4/5). They play essential and various roles in nerve regeneration (Chan et al., 2001). Following nerve injury, Schwann cells promote neuronal cell survival by upregulating GDNF, BDNF, NT-3, NGF and vascular endothelial growth factor (VEGF). While all identified neurotrophins can bind to the p75^NTR^ receptor, their interaction with the TRK family of tyrosine kinase receptors is more selective, each of which prefer particular neurotrophins. NGF exerts its effects primarily through TrkA, while BDNF and NT-4/5 exhibit a higher affinity for TrkB, and NT-3 acts through TrkC (Xu et al., 2023). TrkC/ERK/c-Jun signalling is found to be responsible for the stimulation of nerve regeneration by NT-3 after chronic denervation. In mouse models and patients with Charcot-Marie-Tooth disease type 1A (CMT1A), an inherited peripheral nerve disorder, NT-3 promoted nerve regeneration and improved sensory function (Sahenk et al., 2014). It has been shown that recombinant NT-3 peripheral therapy is safe and well tolerated in Phase I and II clinical trials. Thus, NT-3 may be a potential therapeutic target for PNI (Sahenk et al., 2014; Chaudhry et al., 2000).

#### 2.1.4 Brain derived neurotrophic factor (BDNF)

BDNF is one of the most extensively studied neurotrophic factors, which plays an essential role in peripheral nerve development and regeneration. Numerous studies conducted on various PNI models support the involvement of neurotrophins in the repair and regeneration of peripheral nerve lesions. It has been shown that BDNF mRNA levels are upregulated in DRG and Schwann cells following a nerve injury (McGregor and English, 2018; Yamauchi et al., 2004). Furthermore, BDNF improved myelination through the neurotrophin receptor, p75^NTR^. The interaction between BDNF and p75^NTR^ inhibited Schwann cell migration significantly, with the inhibition of BDNF-induced Schwann cell migration being abolished when p75^NTR^ was knocked down. On the other hand, TrkC receptors play a crucial role in inhibiting NT-3, and blocking these receptors has shown to boost myelin formation. Thus, the data indicates that p75^NTR^ and TrkC receptors exert contrasting effects on myelination (Yamauchi et al., 2004).

Additionally, C3 exoenzyme, Rho inhibitors, and Y-27632, an inhibitor of RhoA effector Rho-kinase, hindered the migration-inhibitory effect of BDNF, indicating that BDNF could inhibit Schwann cell migration via RhoA signalling (Imamura et al., 2000).

#### 2.1.5 Fibroblast growth factor (FGF)

Fibroblast growth factor (FGF) belongs to a family of structurally related heparin-binding growth factors, which have been observed to maintain various biological processes, including promoting the migration of Schwann cells. The ability of therapeutic FGF to regenerate peripheral nerves has been studied in various rodent models (Li et al., 2021; Lu et al., 2019). In a rodent crush injury model, the injection of FGF-21 intramuscularly resulted in an accelerated proliferation of Schwann cells and increased thickness of the myelin sheath (Lu et al., 2019). In primary Schwann cell cultures obtained from rat sciatic nerve, exogenous treatment of platelet-derived growth factor (PDGF) and FGF significantly enhanced cell growth by amplifying DNA synthesis and cell expansion. Furthermore, this response was found to be dependent on increased intracellular cyclic adenosine monophosphate (cAMP) (Davis and Stroobant, 1990).

A later study demonstrated that a specific subtype of FGF, namely the FGF5 ligand, is significantly upregulated in mouse Schwann cells after injury, with fibroblast growth factor receptor (FGFR) 1 and FGFR2 showing increased expression in the Schwann cells of the distal sciatic nerve (Chen et al., 2020). Additionally, primary rat Schwann cell cultures treated with FGF5 presented with increased Schwann cell migration and adhesion rapidly through the upregulation of N-cadherin (Chen et al., 2020).

#### 2.1.6 Transforming growth factor beta-1 (TGF-β1)

TGF-β1 is a multifunctional cytokine that controls proliferation, differentiation, and immune regulation. Several studies indicate that TGF-β1 negatively affects Schwann cell proliferation and differentiation during physiological conditions (Einheber et al., 1995). However, following nerve injury, TGF-β1 has been shown to promote Schwann cells towards a proliferative, non-myelination phenotype, thereby augmenting regenerative responses (Einheber et al., 1995).

TGF-β1 influenced the co-culture of neurons and Schwann cells differently compared to isolated Schwann cells. Primary Schwann cell cultures treated with TGF-β1 have increased cell proliferation and the formation of pre-myelinating or non-myelinating Schwann cells. This phenotype was characterised by an increase in NCAM expression, reduction of nerve growth factor receptor expression as well as the initiation of Oct-6 expression. Additionally, TGF-β1 treatment suppressed the forskolin-mediated induction of P0 and stimulated the Schwann cell transcription factor that suppresses the cAMP inducible POU protein (nomenclature derived from pituitary-specific transcription factor 1 (Pit-1), Octamer-bind protein 1 (Oct-1) and 2 (Oct-2) and the *C. elegans* gene *Unc-86*) (Schonemann et al., 1998). Interestingly, these effects are reversed after TGF-β1 treatment is applied to Schwann cell and neuron co-cultures, with proliferation and myelination being significantly inhibited on contact with the neurons (Einheber et al., 1995).

Additionally, research has found that TGF-β1 significantly induces migration and invasion in a RSC96 Schwann cell line model (Muscella et al., 2020). The TGF-β1-induced migration and invasion were halted by inhibiting matrix metalloproteinase-2 (MMP-2) and matrix metalloproteinase-9 (MMP-9). Furthermore, the migration and invasion effects induced by TGF-β1 were completely abrogated following the application of SB431542, a selective TGF-β1 inhibitor. These findings suggests that TGF-β1 regulates the migration and invasion of RSC96 Schwann cells via MMP-2 and MMP-9 mechanisms. Knockdown of mothers against decapentaplegic homolog 2 (SMAD2), also significantly suppressed the migration and invasion induced by TGF-β1. This observation suggests that SMAD2 could regulate MMP-2. Moreover, TGF-β1 prompted the phosphorylation of ERK1/2 and JNK1/2 and the knockdown of p65/NF-κB restricted the effects of TGF-β1 on MMP-9 and cell migration and invasion. This evidence suggests that MMP-9 could be regulated through the ERK1/2-JNK1/2-NF-κB pathway.

### 2.2 Vitamins and nutrients

#### 2.2.1 Retinoic acid (vitamin A)

Retinoic acid (RA, Vitamin A) was shown to exhibit various roles on Schwann cells in physiology and pathology conditions. Their effects were demonstrated to be mediated through binding to either retinoic acid receptors (RARs) or retinoid X receptors (RXRs). Immunohistochemical analysis following a rat sciatic nerve crush injury model, confirmed the expression of retinoid receptors in Schwann cells (Zhelyaznik and Mey, 2006). Following a nerve crush or transection injury, the RARs were activated, and the local production of RA was elevated, indicating their essential role in peripheral nerve regeneration. Along with the presence of RARs and RXRs in the rat sciatic nerve, the three subtypes of RA synthesizing enzymes retinaldehyde dehydrogenase (RALDH)-1, RALDH-2, and RALDH-3 and the cellular retinoid binding proteins; cellular retinol-binding protein (CRBP)-1, cellular retinoic acid-binding protein (CRABP)-1 and CRABP-2 were detected (Zhelyaznik et al., 2003). The transcript and protein analysis of rat sciatic nerve tissue following a crush and transection injury also showed a 10-fold increase in CRBP-1, facilitating RA synthesis. These studies also showed 15-fold elevations of CRABP-2, an enzyme that might facilitate RA transfer to its nuclear receptors. RA production is enhanced following injury which also enhances Schwann cell migration. This is coupled with the upregulation of neural precursor cell expressed developmentally downregulated protein 9 (NEDD9), a member of the Crk-associated substrate family. The RA-induced NEDD9 increase is attributed to elevated mRNA levels, and NEDD9 protein stability (Latasa et al., 2016). Interestingly, the inactivation of NEDD9 in Schwann cells did not affect basal migration ability, but instead blocked RA-induced migration.

An *in vitro* study using primary Schwann cells, found that post-RA treatment, the transcript concentration of erbB3, which stimulates Schwann cells proliferation and migration, was induced (Zhelyaznik and Mey, 2006). The upregulation of neuregulin (NRG1)-β mRNA at neuromuscular junctions is driven by various regulatory factors such as NT-3, NT-4, BDNF, and GDNF. These factors are also recognized as targets for RA’s transcriptional influence. Besides the RA itself, RA-signalling could be modulated by other factors such as oestrogen, interleukin (IL)-1β, NGF and β-carotene (Scheibe and Wagner, 1992; Mezquita et al., 2018; Lavudi et al., 2023).

#### 2.2.2 Calcitriol (vitamin D3)

Vitamin D3 signalling may contribute to peripheral nerve regeneration through Schwann cells. The vitamin D receptor (VDR), a subclass of nuclear receptors, interacts with the activated form of vitamin D, which is produced by the enzymes vitamin D3-25-OHase and 25-OHD3-1-OHase (Magnaghi et al., 2009; Sakai et al., 2015). The VDR heterodimerizes with the retinoid X receptor (RXR) once activated by the vitamin D ligand, regulating multiple genes containing the vitamin D response element. Several genes involved in myelination, Ca^+2^ homeostasis, proliferation, and differentiation are targeted by VDR. A reduction in myelination in the peripheral nervous system was observed in VDR knockout (KO) mice. Whereas treatment of Schwann cells with Vitamin D analogue showed an improvement in mRNA expression of the myelin basic proteins (MBP), myotrophic and neurotrophic factor, IGF-1. Thus, Schwann cells appear to be affected by vitamin D3.

#### 2.2.3 Ascorbic acid (vitamin C)

Ascorbic acid (Vitamin C), an antioxidant, plays a significant role in various physiological processes. Among its many functions, ascorbic acid facilitates the assembly of basal lamina, Schwann cell differentiation, and myelin formation, partly, through its epigenetic role in ten-eleven translocated enzymes (TET)-mediated DNA demethylation (Huff et al., 2021). It was evident using a co-culture model the neurons failed to become myelinated without ascorbic acid. Furthermore, ascorbic acid was found to reduce cAMP-dependent peripheral myelin protein 22 (PMP22) expression in a dose-dependent manner, likely through the inhibition of adenylate cyclase activity (Kaya et al., 2007). In light of these findings, a clinical trial with ascorbic acid has been initiated for CMT1A (Verhamme et al., 2009). One year of an oral high dose of ascorbic acid was found to be safe but resulted in no significant difference in the motor nerve conduction velocity of the median nerve between the ascorbic acid treated group and the placebo group (Verhamme et al., 2009).

#### 2.2.4 Transferrin (iron)

Transferrin (iron) is an essential element that regulates various processes, including myelin formation and maintenance, DNA synthesis and repair, oxygen transport, and electron transport (Levi and Taveggia, 2014; Salis et al., 2012; El et al., 2021). Researchers found reduced iron storage and incorporation suppressed myelination *in vitro* and *in vivo* (Santiago Gonzalez et al., 2019). Previous work showed that treatment with activating transcription factor (ATF) in an iron-enriched medium prevents Schwann cells reprogramming (Salis et al., 2012). This was evident by the elevated levels of MBP and P0, which are markers for myelin-forming Schwann cells. Conversely, there was a decrease in the indicative marker of Schwann cell precursors, immature Schwann cells, and non-myelinating Schwann cells such as GFAP and p75^NTR^. In rat primary Schwann cell cultures, both ferric ammonium citrate (FAC) and holotransferrin (hTf) were found to encourage differentiation. This promoted differentiation may be attributed to increased cAMP levels, cyclic adenosine monophosphate response element-binding protein (CREB) phosphorylation, and reactive oxygen species (ROS) levels. In contrast, these effects were inhibited when cAMP-PKA antagonists, deferoxamine, and N-acetylcysteine were employed (Salis et al., 2012).

### 2.3 Synthetic compounds

#### 2.3.1 Trichostatin A (TSA)

Trichostatin A (TSA), a natural derivative of dienohydroxamic acid, is known to play various functions including encompassing anti-inflammatory and antioxidant properties and potentially regulating diabetes. It was shown that a high dose of TSA enhanced functional and histological regeneration in the sciatic nerves of diabetic mice (An et al., 2021). After TSA treatment the diabetes-induced BDNF suppression in these mice was effectively reversed (An et al., 2021). TSA treatment of cultured RSC96 Schwann cells in high glucose conditions also significantly triggered BDNF expression. The expression of glucose-regulated protein (GRP)78 and the binding affinity of GRP78 with BDNF has also been enhanced post-TSA treatment. Under high glucose conditions, TSA stimulates the LC3-II/LC3-I ratio and enhances cell autophagy (Shao et al., 2016). Based on this finding, TSA could represent a promising therapeutic target for diabetic peripheral neuropathy.

### 2.4 Hormones and endocrine modulators

#### 2.4.1 17β-estradiol

17β-estradiol, also known as estradiol or E2, is the primary female sex hormone in both humans and rodents. Evidence shows that E2 is involved in both central nervous system (CNS) and PNS myelination and remyelination (Gu et al., 2018). Research has shown that several pathways are essential for Schwann cell myelination and myelin repair, including PI3K/AKT/mTOR and ERK/MAPK (Magnaghi et al., 2009; Gu et al., 2018). Immunostaining and immunoblotting analysis of cultured Schwann cells revealed that E2 stimulates Schwann cell differentiation through the ERβ-ERK1/2 signalling pathway, without affecting proliferation or migration. Additionally, E2 has been found to enhance remyelination in an ovariectomized rat model with a sciatic nerve crush injury. This enhancement is attributed to an increase in the number of lysosomes in Schwann cells, which subsequently facilitates the trafficking of myelin protein to the cell membrane (Gu et al., 2018).

#### 2.4.2 Progesterone

Progesterone is another endogenous steroid hormone that has been studied in various neurological disorders. Research has shown that progesterone is crucial in initiating the myelination process and accelerating the rate of myelin synthesis (Magnaghi et al., 2009; Desarnaud et al., 1998; Magnaghi et al., 2001). However, the amount of myelin formed is not affected by progesterone. In rat primary Schwann cell cultures, progesterone has been observed to stimulate the P0 promoter and promoter 1 of the peripheral myelin protein PMP22 (Melcangi et al., 2000; Azcoitia et al., 2003). P0 and MBP mRNA levels were elevated in response to this activation, along with PMP22, MBP, and P0 proteins. On the other hand, progesterone does not impact MAG expression. This suggests that progesterone benefits may be limited to compact myelin formation rather than axonal integrity.

#### 2.4.3 Erythropoietin (EPO)

Erythropoietin (EPO) is considered a vital element in the development of red blood cells. However, it has a significant role beyond the hematopoietic system (Lehmann and Hoke, 2010). Different CNS and PNS injury models have shown that EPO prevents neuron apoptosis (Siren et al., 2001). Both EPO and its receptor (EPOR) are found in axons, Schwann cells, and endothelial cells of the PNS (Campana and Myers, 2003). Following nerve damage, EPO mRNA increases in Schwann cells and EPOR mRNA in the lumbar DRG. This finding indicates that exogenous recombinant human EPO (rhEPO) treatment could counteract DRG neuron apoptosis, axonal degeneration, and neuropathic pain caused by spinal crush and chronic sciatic nerve injuries (Campana and Myers, 2003).

Furthermore, EPO protects neurons, reduces inflammation, and promotes peripheral nerve regeneration. EPO expression increases in Schwann cells during chronic constriction injury, leading to their proliferation and migration. EPO triggers this effect by binding to its receptor, which triggers a series of cellular events involving JAK-2 and the ERK/MAPK pathway, both of which are necessary for Schwann cell proliferation (Digicaylioglu and Lipton, 2001). Sciatic nerve function was shown to be improved after EPO treatment in mice a week post-injury (Lehmann and Hoke, 2010; Campana and Myers, 2003). Additionally, EPO facilitates nerve regeneration following neurosurgery. EPO treatment enhances early-stage muscle function recovery in specific nerve repair models. EPO’s therapeutic application, especially in its recombinant form, is therefore becoming more promising.

### 2.5 Ion channels modulators and neuropathic medications

#### 2.5.1 Nimodipine

Nimodipine, a calcium antagonist (Cav1.2), inhibits calcium influx by interacting with α1-calcium channel subunits. In clinical settings, it is used to prevent neurological damage following aneurysmal subarachnoid haemorrhage. In recent research, nimodipine has been shown to have beneficial effects on Schwann cells. In an *in vitro* study using the SW10 Schwann cell line, nimodipine treatment significantly reduced the cytotoxicity of Schwann cells under various stress conditions, including ethanol, sodium chloride and heat treatment (Leisz et al., 2019). Similarly, nimodipine decreased caspase activity under stress conditions including ethanol and increased protein kinase B (AKT) and CREB phosphorylation. The evidence suggests that nimodipine inhibits stress-induced apoptosis in Schwann cells by activating the CREB and AKT pathways and reducing caspase 3 activity.

#### 2.5.2 Gamma -aminobutyric acid (GABA)

Gamma-aminobutyric acid (GABA) is the primary inhibitory neurotransmitter in the CNS and has recently been shown to have a role in the PNS, specifically in Schwann cells (Cherchi et al., 2021). Various subunits of the GABA-A receptor, including alpha2, alpha3, and beta1-3, are expressed by Schwann cells (Magnaghi et al., 2001; Melcangi et al., 2000; Magnaghi et al., 2006). Numerous studies have also detected GABA-B receptors in peripheral axons, autonomic nerve terminals, pig nodose ganglion cells, and the rat DRG (Bowery et al., 1981; Desarmenien et al., 1984; Zagorodnyuk et al., 2002).

It has been shown that PMP22 levels are stimulated when Schwann cells are exposed to Muscimol, a GABA-A agonist, indicating that this protein may be under GABA-A regulation. Whereas, treating Schwann cells with baclofen, a GABA-B agonist, decreased levels of several myelin proteins, such as PMP22 and P0, this was correlated with suppression in cell proliferation and the percentage of Schwann-BrdUrd immuno-positive cells (Magnaghi et al., 2004).

GABA may activate intracellular cAMP signalling in Schwann cells to regulate their activity. Additionally, it could be acting through elevating calcium influx, activating MAPK, and phosphorylating CREB (Obrietan et al., 2002). Schwann cell-produced GABA has been shown to trigger the MAPK-CREB signalling pathway (Obrietan et al., 2002). This excitatory activity of GABA is usually seen in early development and so its activity following nerve damage is unknown.

The interplay between GABA-A and GABA-B receptors intricately influences Schwann cell biology. Furthermore, molecular and morphological changes in peripheral myelin are shown in GABA-B1 KO mice, including an elevation in irregular fibres and PMP22 and P0 expression (Magnaghi et al., 2009). Finally, a GABA-increasing drug, valproic acid, enhanced sciatic nerve regeneration in rats, but pregabalin (a GABA analogue) did not (Whitlock et al., 2007).

#### 2.5.3 Glutamate receptors

Various glutamate receptors have been observed in Schwann cells both *in vivo* and *in vitro*, including N-methyl-d-aspartate (NMDA), α-amino-3-hydroxy-5-methyl-4-isoxazolepropionic acid (AMPA), or kainite (KA) receptors, indicating that Schwann cells may also be maintained by glutamate (Kinkelin et al., 2000; Fink et al., 1999; Carozzi et al., 2008). Glutamate transporter 1 (GLT1) transporters are found in Schwann cells’ cytoplasm in rat sciatic nerves. Satellite cells, DRG neurons, and peripheral myelin express glutamate-aspartate transporters (GLAST), whereas the GLT1 transporter is exclusively located in the myelin layer. Neuronal morphology and survival are negatively affected by excitotoxins released by Schwann cells, including glutamate and D-serine.

#### 2.5.4 Lithium chloride

Lithium chloride, a modulator of GSK-3β, was reported to enhance Schwann cell differentiation. It was suggested to be through the role of GSK-3β in PI3K-Akt signalling (Ogata et al., 2004). Additionally, activating the PI3K pathway using adenoviral vectors results in enhanced myelination *in vitro* and *in vivo* (Ogata et al., 2004).

#### 2.5.5 cAMP analogue

The second messenger, cAMP, is a commonly studied pathway in nerve regeneration and repair. cAMP was shown to play a crucial role in Schwann cell proliferation, differentiation, and myelination (Monje, 2015). The research found that forskolin and db-cAMP, a cAMP activator, upregulated P0, MAG, and transcription factor Oct-6 in rat Schwann cells (Jaegle et al., 2003). Interestingly, myelination was enhanced in the absence of proliferation-promoting growth factors. No myelin differentiation markers were induced in cultured mouse Schwann cells following separated treatments with cAMP or NRG1 (Arthur-Farraj et al., 2011). However, when both compounds were combined, there was a significant induction in the expression of myelin markers, Krox-20 and P0. Studies indicate that cAMP can promote myelination in rat and mouse Schwann cells, but not in humans.

cAMP also stimulates Schwann cell proliferation in the presence of mitogens. Both cholera toxin and dbcAMP, similarly promoted the mitogenic effect in Schwann cells (Rutkowski et al., 1995). Activating this pathway by forskolin can result in the simulation of Schwann cell differentiation to a pre-myelinating state.

#### 2.5.6 Pituitary adenylyl cyclase-activating peptide (PACAP)

It has been shown that pituitary adenylyl cyclase-activating peptide (PACAP), produced by neurons and Schwann cells plays a protective role during nerve injury (Maugeri et al., 2020). PACAP induces its protective effect through autocrine or paracrine mechanisms by promoting remyelination and alleviating inflammatory responses following nerve injury. The inflammation is reduced by inhibiting pro-inflammatory cytokine release and enhancing anti-inflammatory cytokine expression in damaged sciatic nerves.

It has been confirmed in several studies that Schwann cells express both PACAP and its receptors. Moreover, gene studies have revealed high levels of PACAP-related genes in the brain and mature Schwann cells. ADCYAP1R1 genes, which encode PAC1R, are highly expressed in mature Schwann cells. PACAP expression was significantly elevated in both Schwann cells and macrophages following a sciatic nerve injury in mice. This elevated expression of various PACAP receptors, including PAC1, VPAC1, and VPAC2 receptors, may activate MAPK/ERK or PI3K/Akt pathway, which are essential to the remyelination of nerves and the proliferation of Schwann cells (Woodley et al., 2019).

It has been shown that PACAP can enhance myelination genes. Expression of myelin-associated proteins, such as MBP, MAG, and P0, are amplified through the PI3K/Akt signalling pathway, which is also involved in Schwann cell survival. PACAP binding to PAC1R, prompts the production of tissue plasminogen activator (tPA) in rat schwannoma cells, which results in the activation of the Akt/CREB pathway (Castorina et al., 2014). This could further aid nerve regeneration and remyelination.

In addition to promoting remyelination, this peptide controls inflammation and supports nerve regeneration. Likewise, PACAP enhances the expression of anti-inflammatory cytokines in distal nerve explants, including IL-4, IL-10, and IL-13. Furthermore, lipopolysaccharide (LPS) induced positive changes in pro-inflammatory cytokines such as IL-6, IL-18, and TNF in RT4-D6P2T Schwann cells, as well as PACAP release (Musumeci et al., 2018).

#### 2.5.7 Syringic acid (SA)

Syringic acid (SA) is a naturally derived substance that has antioxidant, anti-inflammatory, hepatoprotective and neuroprotective activity (Lin et al., 2019). The proliferation and migration of Schwann cells were enhanced by SA treatment in rat Schwann cells (RSC96 cell line). The expression profile of global miRNAs is also evidently altered after SA treatment, specifically miR-451-5p, which is significantly downregulated. By reducing miR-451-5p levels, Schwann cell proliferation and migration were significantly enhanced, indicating that SA mediated its beneficial effects via miR-451-5p (Lin et al., 2019).

#### 2.5.8 Sildenafil

Sildenafil, an oral vasodilator medication and a potent antagonist of phosphodiesterase type 5 (PDE-5), reduces the catabolism of intracellular cyclic guanosine monophosphate (cGMP) (Wang et al., 2005). The reduction in cGMP catabolism results in the cGMP levels remaining elevated for a longer duration. Previous studies using a mouse model of type 2 diabetes, indicate the supportive role of sildenafil on Schwann cell proliferation (Wang et al., 2015). The study also reported that hyperglycaemia-induced downregulation of cGMP correlates with suppressed BDNF expression and reduced Schwann cell proliferation and migration. However, increasing cGMP levels, either by using sildenafil to block PDE-5 or applying the cGMP analogue Br-cGMP, counteracts hyperglycaemic effects on Schwann cells. This highlights the potential beneficial effects of sildenafil in diabetic peripheral neuropathy, which are mediated by the cGMP/PKG signalling pathway in Schwann cells.

#### 2.5.9 Capsaicin

Capsaicin, the active ingredient in chili peppers, is used in topical pain management for its analgesic properties. Previous research has demonstrated that capsaicin exhibits immunomodulatory and antioxidative effects in monoculture Schwann cells (Gruter et al., 2020). This effect was mediated by downregulating the interferon gamma-induced MHC-II production, along with reduced expression of intercellular adhesion molecule 1 mRNA expression and toll-like receptor 4. Moreover, capsaicin induced the resistance of Schwann cells against oxidative stress. However, the direct effect of capsaicin on myelination has not yet been established.

#### 2.5.10 Neurotropin® (NTP)

Neurotropin® (NTP) is a medicinal drug widely prescribed as an analgesic for neuropathic pain in Japan and China (Zhou et al., 2024). This drug is extracted from rabbit skin that has been inflamed by the vaccinia virus. In primary Schwann cells, NTP induced the activity of protein kinase B (AKT), Krox 20 and the expression of MBP and P0 under differentiation conditions (Matsuoka et al., 2018). In contrast, ERK1/2 activity was inhibited in response to NTP. In a DRG and Schwann cell co-culture model, NTP was found to accelerate the myelination process. Histological analysis revealed that NTP promoted remyelination *in vivo* following focal demyelination induced by LPC. Considering these data, NTP appears promising for treating peripheral nerve damage and demyelinating conditions.

#### 2.5.11 Dexamethasone

Dexamethasone, a corticosteroid medication commonly prescribed to reduce inflammation and modulate immune responses in various medical conditions, has also been shown to play a role in peripheral nerve repair (Feng and Yuan, 2015). Research has revealed that dexamethasone stimulates Schwann cell proliferation and promotes the regeneration of myelinated nerve fibres. Additionally, it has been found that dexamethasone treatment improved sciatic functional index as well as sensory nerve conduction velocity and stimulated BDNF expression in injured nerves. The findings indicate that dexamethasone facilitates peripheral nerve regeneration by increasing the expression of BDNF in a rat sciatic nerve injury model (Feng and Yuan, 2015).

#### 2.5.12 Fingolimod

Fingolimod is a sphingosine-1-phosphate (S1P) receptor agonist and FDA-approved treatment for multiple sclerosis. Recent studies indicate that fingolimod treatment activates several Schwann cells reprogramming markers, including c-Jun. Fingolimod also improved the proliferation and migration of Schwann cells (Barber et al., 2004; Heinen et al., 2015). A previous study showed that fingolimod stimulated both neonatal and adult Schwann cells, triggering regeneration-promoting phenotypes. In addition to this shift towards a reprogramming state, fingolimod treated Schwann cells also expressed more growth factors in DRG cultures, which enhanced neurite outgrowth even on inhibitory substrates (Heinen et al., 2015). Hence, these findings suggest that fingolimod should be explored further for PNS regeneration treatments based on S1P receptor stimulation.

#### 2.5.13 Chinese herbal medicines

Numerous studies have shown that various Chinese herbal medicines suppress oxidative stress induced apoptosis in Schwann cells (Wang et al., 2023). High glucose-induced ROS is produced due to mitochondrial oxygen consumption, mitochondrial damage, and nitric oxide (NOX) activation. As a result of ROS upregulation, oxidative stress is triggered, altering several signalling pathways, including the activation of P38 MAPK, AMPK/PGC-1, caspase cascade, suppression of P13K/AKT and separation of Nrf2/Keap1. All of which eventually lead to Schwann cells apoptosis.

Various compounds are known to prevent oxidative stress-induced apoptosis through mitochondrial apoptosis pathways in Schwann cells, such as ginsenoside Rb1, salvia phenolic acids B (Jia et al., 2019), baicalein (Park et al., 2019) and puerarin (Wu et al., 2012).

It has been reported that loganin blocks ROS production (Cheng et al., 2020), while achyranthes bidentata peptides elevate Bcl-2 expression and reduce Bax expression and caspase-3 cleavage, potentially via PI3K/AKT signalling pathway activation. Paeoniflorin may also inhibit Schwann cell apoptosis via the inhibition of the p38 MAPK pathway, whereas baicalein affects apoptosis through the induction of HO-1 expression. The antioxidant properties of paeoniflorin, salvia phenolic acids A, Tang-Luo-Ning decoction extract, and baicalein on Schwann cells could be attributed to their stimulation of Nrf2 expression. In addition, fuzi polysaccharide (Fuzi-p) inhibits ROS accumulation by activating the AMPK-PGC-1-alpha pathway, thereby decreasing apoptosis (Wang et al., 2016).

## 3 Conclusion

In conclusion, this review has highlighted the extensive range of therapies that have potential in modulating Schwann cell proliferation, differentiation, migration, or myelination. An additional benefit is that most of these therapies are already used for other clinical indications, meaning their safety profiles are well established. Repurposing these therapies could enable more rapid translation to the clinic.

Despite multiple studies providing evidence that these therapies have an effect on Schwann cell phenotype or behaviour; this doesn’t always correspond to a beneficial effect on nerve regeneration and functional recovery. Further development and exploration of their mechanistic properties could identify a therapy that is efficacious in treating nerve damage.

Recent studies have recognised the role nerves have in enabling tumour progression and the relationship between them is supported by molecular pathways related to nerve growth and repair. Like in PNI, Schwann cells adopt a de-differentiated phenotype to enable cancer progression (Deborde et al., 2022). Leveraging knowledge and comparing what is known between Schwann cell-mediated repair and nerve invasion by cancer cells is mutually beneficial for both fields and could help identify novel directions for future research and move us closer to developing successful therapies.
